# 3-(4-Bromo­phen­yl)cyclo­pent-2-en-1-one

**DOI:** 10.1107/S160053681401071X

**Published:** 2014-05-21

**Authors:** Endrit Shurdha, Kelsey Dees, Hannah A. Miller, Scott T. Iacono, Gary J. Balaich

**Affiliations:** a Department of Chemistry and Chemistry Research Center, 2355 Fairchild Drive, Suite 2N 225, United States Air Force Academy, Colorado Springs, CO 80840, USA

## Abstract

In the title compound, C_11_H_9_BrO, the cyclo­pentenone ring is almost planar with an r.m.s. deviation of 0.0097 Å. The largest inter-ring torsion angles [2.4 (3), 1.3 (3) and 3.53 (2)°] reveal only a very small twist between the rings, and suggest that the two rings are conjugated. The mol­ecule is slightly bowed, as shown by the small dihedral angle between the rings [5.3 (1)°]. The crystal packing pattern consists of parallel sheets that stack parallel to the *ac* plane. Each sheet consists of mol­ecules that pack side-to-side with the same relative orientation of phenyl and cyclo­pentenone rings along the *a*- and *c*-axis directions. Slipped side-to-side, face-to-face and edge-to-face inter­actions exist between pairs of sheets with edge-to-edge and edge-to-face O⋯H—C(*sp* ^2^) weak hydrogen-bond contacts. A relatively short edge-to-face contact (2.77 Å) also exists between pairs of sheets.

## Related literature   

For structures of related 3-Ph substituted cyclo­pent-2-ene-1-ones, see: Zhao *et al.* (2008 ▶); Marjani *et al.* (2007 ▶, 2008 ▶); Jedrzejas *et al.* (1996 ▶). For leading references on the synthesis and uses of substituted cyclo­pentenones, see: Gibson *et al.* (2004 ▶); Gibson & Mainolfi (2005 ▶); Liu *et al.* (2013 ▶); Barluenga *et al.* (2012 ▶); Varea *et al.* (2012 ▶). For materials chemistry applications, see: Peloquin *et al.* (2012 ▶); Li *et al.* (2008 ▶). For the synthesis of the title compound, see: Heck (1965 ▶). For weak hydrogen bonds, see: Arunan *et al.* (2011 ▶).
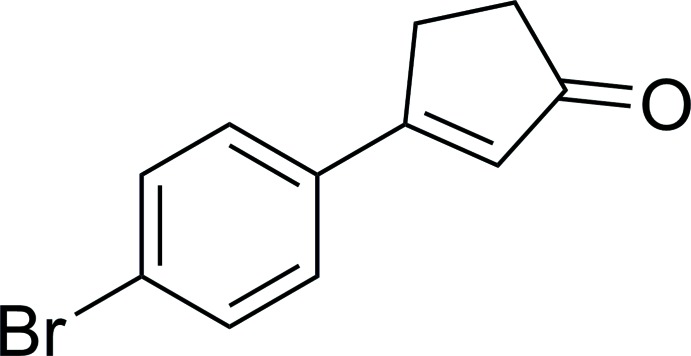


## Experimental   

### 

#### Crystal data  

C_11_H_9_BrO
*M* *_r_* = 237.09
Monoclinic, 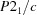

*a* = 10.0219 (12) Å
*b* = 9.7818 (11) Å
*c* = 9.9945 (12) Å
β = 107.4375 (14)°
*V* = 934.76 (19) Å^3^
*Z* = 4
Mo *K*α radiation
μ = 4.35 mm^−1^
*T* = 100 K
0.30 × 0.13 × 0.07 mm

#### Data collection  

Bruker SMART APEX CCD diffractometer
Absorption correction: multi-scan *SADABS* (Bruker, 2013 ▶) *T*_min_ = 0.53, *T*_max_ = 0.75
9994 measured reflections
2316 independent reflections
1917 reflections with *I* > 2σ(*I*)
*R*_int_ = 0.031

#### Refinement  

*R*[*F* ^2^ > 2σ(*F* ^2^)] = 0.025
*wR*(*F* ^2^) = 0.057
*S* = 1.02
2316 reflections
118 parameters
H-atom parameters constrained
Δρ_max_ = 0.35 e Å^−3^
Δρ_min_ = −0.46 e Å^−3^

### 

Data collection: *APEX2* (Bruker, 2013 ▶); cell refinement: *SAINT* (Bruker, 2013 ▶); data reduction: *SAINT*; program(s) used to solve structure: *SHELXS2013* (Sheldrick, 2008 ▶); program(s) used to refine structure: *SHELXL2013* (Sheldrick, 2008 ▶); molecular graphics: *SHELXP* (Sheldrick, 2008 ▶) and *Mercury* (Macrae *et al.*, 2008 ▶); software used to prepare material for publication: *SHELXL2013* (Sheldrick, 2008 ▶).

## Supplementary Material

Crystal structure: contains datablock(s) global, I. DOI: 10.1107/S160053681401071X/mw2122sup1.cif

Structure factors: contains datablock(s) I. DOI: 10.1107/S160053681401071X/mw2122Isup2.hkl

Supporting information file. DOI: 10.1107/S160053681401071X/mw2122Isup3.cdx

Supporting information file. DOI: 10.1107/S160053681401071X/mw2122Isup4.cml

CCDC reference: 999140

Additional supporting information:  crystallographic information; 3D view; checkCIF report

## Figures and Tables

**Table 1:** Hydrogen-bond geometry (Å, °)

*D*—H⋯*A*	*D*—H	H⋯*A*	*D*⋯*A*	*D*—H⋯*A*
C2—H2⋯O1^i^	0.95	2.58	3.465 (2)	154
C7—H7⋯O1^i^	0.95	2.58	3.484 (3)	158
C10—H10⋯O1^ii^	0.95	2.52	3.377 (2)	150

Symmetry codes: (i) 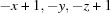; (ii) 
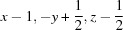
.

**Fig. 1.**
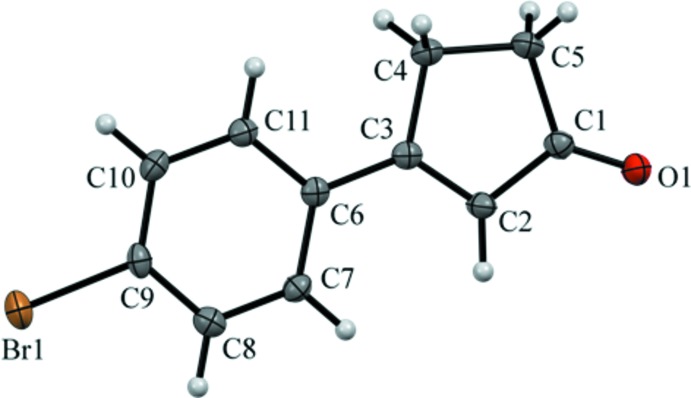
Molecular structure of 3-(4-bromophenyl)cyclopent-2-en-1-one. Ellipsoids are shown at the 50% probability level.

**Fig. 2.**
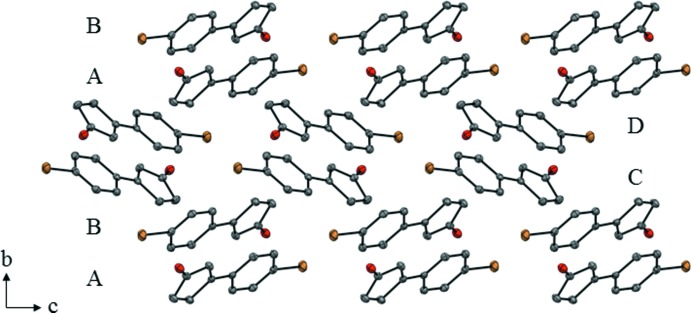
Crystal packing view of 3-(4-bromophenyl)cyclopent-2-en-1-one. View is down the a axis, and shows sheets A-D which stack parallel to the ac plane. Ellipsoids are shown at the 50% probability level. Hydrogen atoms were omitted for clarity.

**Fig. 3.**
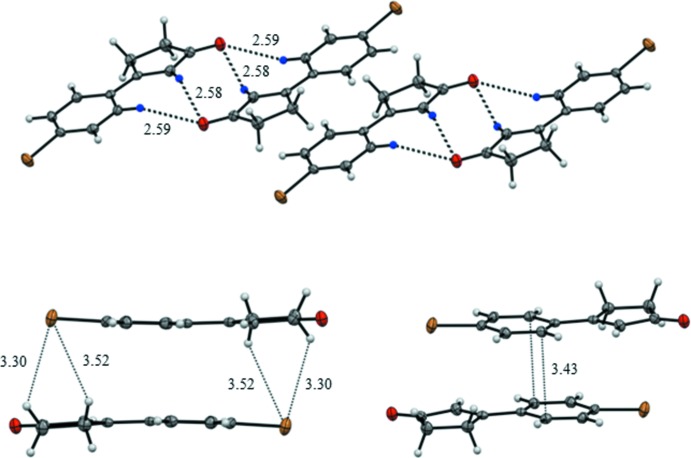
Molecular packing motifs depicting side-to-side and face-to-face intermolecular contacts. The bottom left diagram depicts the bowed molecular axis. The bottom right diagram indicates the C6–Ph ring centroid distance (3.43 Å), which is within error of the C7–C11 distances (3.42 Å, dashed lines). Ellipsoids are shown at the 50% probability level. Distances shown are in Å.

## supplementary crystallographic information

### 1. Comment

Substituted cyclo­pentenones are found most frequently as inter­mediates for or parts of complex bioactive molecules and are synthesized by a variety of metal- and non-metal-mediated methodologies (Liu *et al.* (2013), Barluenga *et al.* (2012), Varea *et al.* (2012), Gibson *et al.* (2005), Gibson *et al.* (2004)). We are inter­ested in the use of substituted cyclo­pentenones as inter­mediates in the synthesis of fulvenes or fulvene-based polymers with optoelectronic properties suitable for possible molecular electronics applications (Peloquin *et al.* (2012)). The title compound was targeted due to the importance of the Br substituent in Sonagashira or Suzuki coupling methods which will be used to extend the π conjugation of the cyclo­pentenone and resulting fulvene molecular frameworks. In the course of purifying the title compound, crystals were obtained and, as its structure had not been published, its structure was determined.

The title compound (Fig. 1) consists of a planar Ph ring and an almost planar cyclo­pentenone ring with r.m.s deviations from the least squares planes of 0.0062 Å (Ph) and 0.0097 Å (cyclo­pentenone). Conjugation of both rings is evident from the small torsion angles about the C3—C6 bond (C2C3C6C7 = 2.4 (3)°, C4C3C6C11 = 1.3 (3)° and C4C3C6C7 = 3.53 (2)°). The dihedral angle between the phenyl and cyclo­pentenone rings is 5.3 (1)° resulting in the molecule being slightly bowed along its long axis.

The crystal packing pattern consists of parallel sheets of cyclo­pentenone molecules (sheets A—D, Fig. 2) that stack parallel to the *ac* plane. Each sheet consists of molecules that pack side-to-side with the same relative orientation of Ph and cyclo­pentenone groups along the a- and c-axis directions. Slipped side-to-side, face-to-face and edge-to-face inter­actions exist between pairs of sheets AB, CD, BC, and AD. The shortest inter­molecular contacts are weak O···H—C(sp^2^) hydrogen bonds (Table 1) with molecules in edge-to-edge and edge-to-face orientations. Although the D—H···A angles (Table 1) deviate substanti­ally from the 180° expected for strong hydrogen bonds, they are large enough to be classified as weak hydrogen bonds (Arunan *et al.* (2011). Furthermore, the H···A distances are shorter than the sum of the H and O van der Waals radii (2.72 Å) (Arunan *et al.* (2011)). The shortest inter­molecular contact besides the noted weak hydrogen bonds is the edge-to-face C11–H8 distance (2.77 Å).

### 2. Experimental

The synthesis of the title compound was carried out using a modification to the original literature procedure (Heck (1965)). The diketone, 1-(4-bromo­phenyl)-1,4-pentane­dione (10.0 g, 39.2 mmol) was combined with 0.5 M NaOH (1 L) and the reaction mixture vigorously stirred and heated at 90 °C for 4 hours. Aliquots were periodically removed to monitor the progress of the reaction by MS-TOF. The reaction mixture was allowed to cool and subsequently neutralized with 1M H_2_SO_4_. The resulting precipitate was collected by vacuum filtration, washed with water (100 mL), and vacuum dried to give a light brown crude product. Purification was best achieved by column chromatography (dry loaded from CH_2_Cl_2_) using an EtOAc/hexane mobile phase. A mobile phase of 10% EtOAc/hexane was used to initially remove the colored impurities. This process was followed by 20% EtOAc/hexane to obtain 3-(4-bromo­phenyl)­cyclo­pent-2-en-1-one as a yellow solid (Yield 5.85 g, 63%). ^1^H NMR (CDCl_3_) d 2.55, 2.98 (m, 4H, CH_2_); 6.52 (t, 1H, CHCO, ^4^J = 2 Hz); 7.48, 7.55 (m, 4H, BrPhH). ^13^C NMR (CDCl_3_) d 28.5, 35.2 (CH_2_); 125.6, 127.8, 128.1, 132.1, 132.9 (CH and C); 172.3 (CBr); 208.8 (CO). MS-TOF [M+H]^+^calcd. For C_11_H_9_BrO 236.9915; found 236.9927.

### 3. Refinement

All hydrogen atoms were placed in calculated positions using a riding model (aryl C—H = 0.95 Å, methyl­ene C—H = 0.99 Å; U_iso_(H) = 1.2 U_eq_(C)).

### Crystal data

**Table:**

C_11_H_9_BrO	*D*_x_ = 1.685 Mg m^−^^3^
*M**_r_* = 237.09	Melting point: 127.0 K
Monoclinic, *P*2_1_/*c*	Mo *K*α radiation, λ = 0.71073 Å
*a* = 10.0219 (12) Å	Cell parameters from 3089 reflections
*b* = 9.7818 (11) Å	θ = 3.0–28.6°
*c* = 9.9945 (12) Å	µ = 4.35 mm^−^^1^
β = 107.4375 (14)°	*T* = 100 K
*V* = 934.76 (19) Å^3^	Rectangular prism, colourless
*Z* = 4	0.30 × 0.13 × 0.07 mm
*F*(000) = 472	

### Data collection

**Table:**

Bruker SMART APEX CCD diffractometer	2316 independent reflections
Radiation source: fine focus sealed tube	1917 reflections with *I* > 2σ(*I*)
Graphite monochromator	*R*_int_ = 0.031
Detector resolution: 8.3333 pixels mm^-1^	θ_max_ = 28.3°, θ_min_ = 2.1°
ω scans	*h* = −13→13
Absorption correction: multi-scan *SADABS* (Bruker, 2013)	*k* = −12→13
*T*_min_ = 0.53, *T*_max_ = 0.75	*l* = −13→13
9994 measured reflections	

### Refinement

**Table:**

Refinement on *F*^2^	Primary atom site location: structure-invariant direct methods
Least-squares matrix: full	Secondary atom site location: difference Fourier map
*R*[*F*^2^ > 2σ(*F*^2^)] = 0.025	Hydrogen site location: inferred from neighbouring sites
*wR*(*F*^2^) = 0.057	H-atom parameters constrained
*S* = 1.02	*w* = 1/[σ^2^(*F*_o_^2^) + (0.0233*P*)^2^ + 0.4987*P*] where *P* = (*F*_o_^2^ + 2*F*_c_^2^)/3
2316 reflections	(Δ/σ)_max_ = 0.001
118 parameters	Δρ_max_ = 0.35 e Å^−^^3^
0 restraints	Δρ_min_ = −0.46 e Å^−^^3^

### Special details

**Table:**

Geometry. All e.s.d.'s (except the e.s.d. in the dihedral angle between two l.s. planes) are estimated using the full covariance matrix. The cell e.s.d.'s are taken into account individually in the estimation of e.s.d.'s in distances, angles and torsion angles; correlations between e.s.d.'s in cell parameters are only used when they are defined by crystal symmetry. An approximate (isotropic) treatment of cell e.s.d.'s is used for estimating e.s.d.'s involving l.s. planes.

### Fractional atomic coordinates and isotropic or equivalent isotropic displacement parameters (Å^2^)

**Table:**

	*x*	*y*	*z*	*U*_iso_*/*U*_eq_	
Br1	−0.30717 (2)	0.07926 (2)	0.08100 (2)	0.02888 (8)	
O1	0.59890 (14)	0.08928 (13)	0.72449 (14)	0.0228 (3)	
C1	0.4771 (2)	0.12867 (19)	0.68736 (19)	0.0181 (4)	
C2	0.3642 (2)	0.07755 (19)	0.56825 (19)	0.0181 (4)	
H2	0.3743	0.0067	0.507	0.022*	
C3	0.2442 (2)	0.14462 (17)	0.55725 (18)	0.0154 (4)	
C4	0.2622 (2)	0.24952 (19)	0.67199 (19)	0.0204 (4)	
H4A	0.2025	0.2274	0.732	0.024*	
H4B	0.2377	0.342	0.6319	0.024*	
C5	0.4180 (2)	0.2416 (2)	0.7563 (2)	0.0229 (4)	
H5A	0.4655	0.3295	0.7519	0.027*	
H5B	0.4294	0.2194	0.8558	0.027*	
C6	0.10968 (19)	0.12424 (18)	0.44782 (18)	0.0152 (4)	
C7	0.0981 (2)	0.03111 (18)	0.33801 (19)	0.0177 (4)	
H7	0.1767	−0.0228	0.3368	0.021*	
C8	−0.0265 (2)	0.01702 (19)	0.23155 (19)	0.0198 (4)	
H8	−0.0338	−0.0465	0.1578	0.024*	
C9	−0.1403 (2)	0.09624 (19)	0.23345 (19)	0.0187 (4)	
C10	−0.1341 (2)	0.18783 (19)	0.34090 (19)	0.0191 (4)	
H10	−0.2135	0.2406	0.3416	0.023*	
C11	−0.0086 (2)	0.20035 (18)	0.44772 (19)	0.0181 (4)	
H11	−0.0029	0.2622	0.5225	0.022*	

### Atomic displacement parameters (Å^2^)

**Table:**

	*U*^11^	*U*^22^	*U*^33^	*U*^12^	*U*^13^	*U*^23^
Br1	0.02309 (12)	0.03472 (13)	0.02207 (11)	0.00371 (9)	−0.00349 (8)	−0.00033 (8)
O1	0.0171 (7)	0.0241 (7)	0.0251 (7)	0.0006 (5)	0.0031 (6)	−0.0043 (6)
C1	0.0187 (10)	0.0163 (8)	0.0192 (9)	−0.0016 (7)	0.0054 (8)	−0.0006 (7)
C2	0.0183 (10)	0.0187 (9)	0.0179 (9)	−0.0011 (7)	0.0063 (8)	−0.0043 (7)
C3	0.0189 (9)	0.0134 (8)	0.0157 (8)	−0.0017 (7)	0.0077 (7)	0.0018 (6)
C4	0.0223 (10)	0.0172 (9)	0.0218 (9)	0.0016 (7)	0.0068 (8)	−0.0031 (7)
C5	0.0239 (10)	0.0203 (9)	0.0226 (9)	0.0004 (8)	0.0039 (8)	−0.0071 (8)
C6	0.0168 (9)	0.0135 (8)	0.0159 (8)	−0.0017 (7)	0.0061 (7)	0.0033 (6)
C7	0.0169 (10)	0.0175 (8)	0.0202 (9)	0.0012 (7)	0.0078 (8)	0.0007 (7)
C8	0.0219 (10)	0.0197 (9)	0.0180 (9)	−0.0028 (7)	0.0063 (8)	−0.0013 (7)
C9	0.0167 (10)	0.0215 (9)	0.0161 (9)	−0.0004 (7)	0.0021 (7)	0.0053 (7)
C10	0.0181 (10)	0.0178 (9)	0.0222 (9)	0.0041 (7)	0.0073 (8)	0.0028 (7)
C11	0.0211 (10)	0.0166 (8)	0.0177 (9)	0.0011 (7)	0.0076 (8)	0.0009 (7)

### Geometric parameters (Å, º)

**Table:**

Br1—C9	1.9022 (19)	C5—H5B	0.99
O1—C1	1.226 (2)	C6—C11	1.399 (3)
C1—C2	1.463 (3)	C6—C7	1.404 (3)
C1—C5	1.514 (3)	C7—C8	1.383 (3)
C2—C3	1.346 (3)	C7—H7	0.95
C2—H2	0.95	C8—C9	1.384 (3)
C3—C6	1.473 (3)	C8—H8	0.95
C3—C4	1.509 (2)	C9—C10	1.386 (3)
C4—C5	1.538 (3)	C10—C11	1.391 (3)
C4—H4A	0.99	C10—H10	0.95
C4—H4B	0.99	C11—H11	0.95
C5—H5A	0.99		
			
O1—C1—C2	126.57 (17)	H5A—C5—H5B	108.8
O1—C1—C5	125.55 (17)	C11—C6—C7	118.25 (17)
C2—C1—C5	107.87 (16)	C11—C6—C3	120.99 (16)
C3—C2—C1	110.76 (16)	C7—C6—C3	120.72 (16)
C3—C2—H2	124.6	C8—C7—C6	120.64 (18)
C1—C2—H2	124.6	C8—C7—H7	119.7
C2—C3—C6	126.28 (17)	C6—C7—H7	119.7
C2—C3—C4	111.66 (16)	C7—C8—C9	119.44 (18)
C6—C3—C4	122.05 (16)	C7—C8—H8	120.3
C3—C4—C5	104.63 (15)	C9—C8—H8	120.3
C3—C4—H4A	110.8	C8—C9—C10	121.80 (18)
C5—C4—H4A	110.8	C8—C9—Br1	118.07 (14)
C3—C4—H4B	110.8	C10—C9—Br1	120.11 (14)
C5—C4—H4B	110.8	C9—C10—C11	118.16 (17)
H4A—C4—H4B	108.9	C9—C10—H10	120.9
C1—C5—C4	105.03 (15)	C11—C10—H10	120.9
C1—C5—H5A	110.7	C10—C11—C6	121.68 (17)
C4—C5—H5A	110.7	C10—C11—H11	119.2
C1—C5—H5B	110.7	C6—C11—H11	119.2
C4—C5—H5B	110.7		
			
O1—C1—C2—C3	−179.48 (18)	C4—C3—C6—C7	176.47 (16)
C5—C1—C2—C3	−0.5 (2)	C11—C6—C7—C8	1.1 (3)
C1—C2—C3—C6	177.94 (16)	C3—C6—C7—C8	−176.78 (16)
C1—C2—C3—C4	−1.0 (2)	C6—C7—C8—C9	0.3 (3)
C2—C3—C4—C5	2.0 (2)	C7—C8—C9—C10	−1.3 (3)
C6—C3—C4—C5	−176.98 (16)	C7—C8—C9—Br1	177.38 (14)
O1—C1—C5—C4	−179.30 (18)	C8—C9—C10—C11	0.9 (3)
C2—C1—C5—C4	1.7 (2)	Br1—C9—C10—C11	−177.78 (13)
C3—C4—C5—C1	−2.16 (19)	C9—C10—C11—C6	0.5 (3)
C2—C3—C6—C11	179.84 (18)	C7—C6—C11—C10	−1.5 (3)
C4—C3—C6—C11	−1.3 (3)	C3—C6—C11—C10	176.34 (16)
C2—C3—C6—C7	−2.4 (3)		

### Hydrogen-bond geometry (Å, º)

**Table:**

*D*—H···*A*	*D*—H	H···*A*	*D*···*A*	*D*—H···*A*
C2—H2···O1^i^	0.95	2.58	3.465 (2)	154
C7—H7···O1^i^	0.95	2.58	3.484 (3)	158
C10—H10···O1^ii^	0.95	2.52	3.377 (2)	150

Symmetry codes: (i) −*x*+1, −*y*, −*z*+1; (ii) *x*−1, −*y*+1/2, *z*−1/2.
